# Circulating PDIA4, MMP-2, MMP-9, and Claudin-2 in Vitamin D-Deficient Patients with Brain Tumors: An Exploratory Clinical Pilot Study

**DOI:** 10.3390/jcm15166192

**Published:** 2026-08-10

**Authors:** Bartłomiej Gromadzki, Michał Wiciński, Zygmunt Siedlecki, Rafał Porzych, Igor Pisarski

**Affiliations:** 1 Department of Pharmacology and Therapeutics, Collegium Medicum in Bydgoszcz, Nicolaus Copernicus University in Toruń, ul. Curie Skłodowskiej 9, 85-090 Bydgoszcz, Poland; michal.wicinski@cm.umk.pl (M.W.); igor.pisarski@cm.umk.pl (I.P.); 2 Department of Neurosurgery, Neurotraumatology and Pediatric Neurosurgery, Collegium Medicum in Bydgoszcz, Nicolaus Copernicus University in Toruń, 85-094 Bydgoszcz, Poland; siedlecki@cm.umk.pl

**Keywords:** brain tumors, glioblastoma, brain metastases, PDIA4, matrix metalloproteinases, claudin-2, blood–brain barrier, extracellular matrix, serum biomarkers, vitamin D deficiency

## Abstract

**Background/Objectives:** Extracellular matrix remodeling, cellular stress responses, and blood–brain/blood–tumor barrier-related alterations are important processes involved in brain tumor biology. This exploratory cross-sectional pilot study evaluated circulating circulating protein disulfide isomerase A4 (PDIA4), matrix metalloproteinase-2 (MMP-2), matrix metalloproteinase-9 (MMP-9), and claudin-2 (CLDN2) concentrations in vitamin D-deficient patients with different brain tumor entities and aimed to provide preliminary effect-size estimates for future studies. **Methods:** A total of 62 vitamin D-deficient participants were included, comprising patients with glioblastoma (GBM; *n* = 15), brain metastases (*n* = 20), and meningioma (*n* = 8) and surgical controls with degenerative spine disease (*n* = 19). Serum biomarker concentrations were measured using enzyme-linked immunosorbent assays. Global between-group effect sizes were estimated using η^2^, while exploratory pairwise comparisons and associations with tumor size were assessed using non-parametric methods. Effect-size estimates were prioritized, with *p*-values reported to provide complementary inferential context. **Results:** The largest global between-group effect-sizes were observed for CLDN2 (η^2^ = 0.35) and MMP-2 (η^2^ = 0.22), whereas the estimated effects were small for MMP-9 (η^2^ = 0.05) and negligible for PDIA4 (η^2^ = 0.00). Exploratory pairwise comparisons indicated lower serum MMP-2 concentrations in patients with GBM than in surgical controls and lower CLDN2 concentrations in patients with GBM and brain metastases than in controls. Positive exploratory associations were observed between tumor size and PDIA4 concentration in the GBM group (Spearman’s rho = 0.56; 95% CI: 0.02–0.90) and between tumor size and MMP-2 concentration in patients with brain metastases (rho = 0.46; 95% CI: 0.01–0.90). **Conclusions:** These preliminary effect-size estimates, together with the exploratory correlations with tumor size, may inform biomarker selection and sample-size planning in future prospective studies. The findings should not be interpreted as robust diagnostic or prognostic evidence and require validation in larger, independent, and well-controlled cohorts.

## 1. Introduction

Remodeling of the extracellular matrix (ECM) and disruption of the blood–brain barrier (BBB) and blood–tumor barrier (BTB) are important processes involved in the development, invasiveness, and clinical behavior of brain tumors. These mechanisms may be reflected by circulating biomarkers associated with matrix degradation, cellular stress responses, and barrier-related proteins.

Central nervous system (CNS) tumors comprise a heterogeneous group of diseases, including highly malignant primary tumors such as glioblastoma (GBM), secondary brain metastases, and generally benign but clinically relevant tumors such as meningiomas. These entities differ substantially in biological behavior, invasiveness, vascular remodeling, interactions with the BBB/BTB, and prognosis [1]. GBM is the most common malignant primary brain tumor [2], brain metastases represent the most frequent intracranial tumors in adults [3,4], and meningiomas are among the most common primary intracranial tumors [5]. This biological and clinical heterogeneity makes these tumor types relevant comparative groups for exploratory biomarker studies.

Vitamin D deficiency is common and clinically relevant, particularly in patients with cancer [6,7]. Therefore, studying vitamin D-deficient individuals reflects a real-world clinical scenario rather than an artificially selected or marginal population. Since vitamin D status may influence immune and inflammatory responses, as well as extracellular matrix remodeling [8,9], restricting the study cohort to vitamin D-deficient individuals was intended to reduce biological heterogeneity related to vitamin D status. Thus, vitamin D deficiency was treated as a defining characteristic of the study population rather than as an independent exposure variable.

Protein disulfide isomerase A4 (PDIA4), also known as ERp72, is a member of the protein disulfide isomerase family that participates in protein folding within the endoplasmic reticulum (ER) and in maintaining cellular redox homeostasis [10]. PDIA4 has been implicated in several processes relevant to tumor development and progression. By enhancing vascular endothelial growth factor (VEGF) expression, it may contribute to tumor angiogenesis. In the context of tumor progression, PDIA4 has been associated with malignant cell behavior, including enhanced proliferation, survival, migration, and invasion [11,12]. Malignant cells often experience ER stress due to rapid proliferation, increased metabolic demand, hypoxia, and oxidative stress within the tumor microenvironment. By supporting protein folding and limiting the accumulation of misfolded proteins, PDIA4 may facilitate tumor cell adaptation to these unfavorable conditions and promote cell survival [13,14]. PDIA4 overexpression has been reported in several malignancies and has been associated with tumor progression, resistance to cellular stress, and poorer clinical outcomes [13,14].

In glioblastoma, PDIA4 is overexpressed and facilitates VEGF-A secretion, thereby promoting tumor angiogenesis and enhancing tumor cell survival under ER stress conditions. This mechanism appears to be at least partly dependent on the XBP1 pathway, a component of the unfolded protein response (UPR) [15]. These observations suggest that PDIA4 may be biologically relevant in malignant brain tumors; however, its circulating serum concentrations in different brain tumor entities remain insufficiently characterized.

Matrix metalloproteinase-2 (MMP-2), also known as gelatinase A, is a zinc-dependent endopeptidase involved in the degradation of extracellular matrix components, including type IV collagen and gelatin [16]. In brain tumors, particularly gliomas, MMP-2 has been implicated in tumor invasion, extracellular matrix remodeling, angiogenesis, and BBB/BTB disruption [16,17]. Tissue-based studies have reported increased MMP-2 expression in high-grade gliomas when compared with lower-grade tumors or normal brain tissue, and higher MMP-2 expression has been associated with more aggressive tumor behavior and poorer outcomes [18]. However, whether circulating MMP-2 concentrations reflect these tissue-level processes remains uncertain.

Matrix metalloproteinase-9 (MMP-9), also known as gelatinase B, is another ECM-degrading enzyme involved in tumor invasion, angiogenesis, immune–microenvironment interactions, and vascular permeability [19,20]. In malignant brain tumors, especially glioblastoma, MMP-9 has been linked to basement membrane component degradation, BBB/BTB disruption, peritumoral remodeling, and adverse clinical behavior [19,20,21]. Elevated MMP-9 expression has been observed more frequently in high-grade than in low-grade gliomas and has been associated with shorter survival in several studies [22,23]. Similar to MMP-2, it remains uncertain whether circulating MMP-9 concentrations accurately reflect tumor-associated MMP-9 expression or activity.

Claudin-2 (CLDN2) is a tight-junction protein that forms paracellular channels permeable to cations and water [24]. Beyond its structural role in intercellular junctions, CLDN2 has been implicated in the regulation of cell proliferation, migration, survival, and differentiation [24,25,26]. In several epithelial malignancies, altered CLDN2 expression has been associated with tumor growth, epithelial–mesenchymal transition, and metastatic behavior, although its role appears to be strongly tissue- and context-dependent [24,25,26]. In the central nervous system, CLDN2 is not considered a dominant endothelial tight junction protein of the BBB [27,28]. Data on CLDN2 in brain tumors remain limited, although available studies suggest that its expression may depend on tumor type and differentiation status, including selected CNS tumors such as ependymoma [27,28,29,30]. Therefore, the assessment of serum CLDN2 should be regarded as exploratory. In the present study, CLDN2 was included not as a direct marker of BBB integrity, but as a tight-junction-associated protein that may provide preliminary information on barrier-related or tumor-type-specific biological alterations.

The primary objective of this exploratory pilot study was to assess circulating serum concentrations of PDIA4, MMP-2, MMP-9, and CLDN2 in vitamin D-deficient patients with different brain tumor entities, including glioblastoma, brain metastases, and meningioma, compared with a surgical control group. The secondary objectives were to evaluate exploratory associations between these circulating biomarkers and tumor size and to provide preliminary global between-group effect-size estimates that could inform the design and sample-size planning of future adequately powered biomarker studies.

## 2. Materials and Methods

### 2.1. Bioethics

In accordance with the Declaration of Helsinki, this study received approval from the Bioethics Committee of the Collegium Medicum in Bydgoszcz, Nicolaus Copernicus University, Toruń, Poland (approval number: KB 115/2021; date of approval: 16 February 2021). Written informed consent was obtained from all participants prior to their inclusion in this study.

This study was observational and non-interventional in nature. Participants were not allocated to any therapeutic intervention, and blood sampling was performed in the perioperative setting; therefore, prospective clinical trial registration was not required.

### 2.2. Study Cohort

A total of 62 patients from the Department of Neurosurgery at the Specialist City Hospital in Toruń were enrolled in this study. Patients were selected from individuals undergoing neurosurgical treatment during the study period based on predefined eligibility criteria, including vitamin D deficiency and availability of preoperative serum samples. Tumor patients were subsequently classified into diagnostic groups according to postoperative histopathological diagnosis. The study cohort therefore represents a surgically treated neurosurgical population rather than the entire spectrum of patients with brain tumors. In particular, patients with GBM who were not candidates for surgical treatment were not represented, which may introduce selection and survivorship bias. A simplified patient selection and diagnostic classification diagram is provided as Supplementary Figure S1. The experimental cohort comprised 43 patients who underwent surgical treatment for brain tumors and was further stratified into three subgroups according to diagnosis: glioblastoma multiforme (GBM, WHO grade IV), brain metastases, and meningioma. All participants were vitamin D-deficient. Vitamin D deficiency was defined as a serum 25-hydroxyvitamin D [25(OH)D] concentration below 20 ng/mL (<50 nmol/L), according to commonly used clinical criteria. The control group included 19 individuals without cancer, who were non-smokers, free from metabolic disorders, and undergoing neurosurgical procedures for spinal disk disease. This study was conducted between September 2021 and February 2022 at the Department of Neurosurgery, Specialist City Hospital, Toruń, where blood samples were collected from all participants for subsequent analyses.

### 2.3. Measurements

Venous blood samples were obtained from all participants. The specimens were processed according to standard laboratory procedures, which included serum preparation, freezing at −20 °C, and transport on dry ice to the Department of Pharmacology and Therapeutics, Collegium Medicum, Bydgoszcz, where they were stored at −70 °C until analysis. The measured parameters—PDIA4, MMP-2, MMP-9, and CLDN2—were quantified using enzyme-linked immunosorbent assays (ELISAs) following the manufacturer’s protocols (Sunredbio [SRB] Technology, Shanghai, China). All assays were performed using an EPOCH microplate spectrophotometer (BioTek, Santa Clara, CA, USA).

### 2.4. Statistical Analysis

Data are presented as median (interquartile range, IQR) or mean (standard deviation, SD). The distribution of variables was assessed using the Shapiro–Wilk test. Given the non-normal distribution of the data, between-group analyses were performed using the Kruskal–Wallis test followed by post hoc pairwise comparisons. For each biomarker, the effect size associated with the Kruskal–Wallis test was estimated using η^2^ = (H − k + 1)/(*N* − k), where H is the Kruskal–Wallis statistic, k is the number of groups, and *N* is the total sample size; negative values were set to zero. In accordance with the exploratory pilot design, the interpretation focused primarily on effect-size estimates, while test statistics and *p*-values were reported to provide complementary inferential context. The Benjamini–Hochberg procedure was applied to post hoc pairwise comparisons. Spearman’s rank correlation coefficient was employed to evaluate associations between different parameters. Confidence intervals for correlation coefficients were estimated using bootstrap resampling. All statistical analyses were carried out using STATISTICA version 13.3 (TIBCO Software Inc., Palo Alto, CA, USA).

## 3. Results

A total of 62 vitamin D-deficient participants were included in the analysis. The study population consisted of patients with glioblastoma WHO grade IV (GBM; *n* = 15), patients with brain metastases (*n* = 20), patients with meningioma (*n* = 8), and a control group composed of patients undergoing neurosurgical procedures for degenerative spine disease (*n* = 19). Table 1 shows the numbers in each group, sex distribution per group, age, and vitamin D3 concentrations. No clear between-group differences were observed in the assessed baseline characteristics.

Normality testing showed that the distribution of several analyzed variables deviated from normality within individual study groups. Therefore, non-parametric statistical methods were used for between-group comparisons and correlation analyses. The largest global between-group effect-size estimates were observed for CLDN2 (η^2^ = 0.35) and MMP-2 (η^2^ = 0.22), whereas smaller estimates were observed for MMP-9 (η^2^ = 0.05) and PDIA4 (η^2^ = 0.00). The corresponding Kruskal–Wallis test statistics and *p*-values are presented in Table 2 as complementary inferential context.

**Figure 1:**
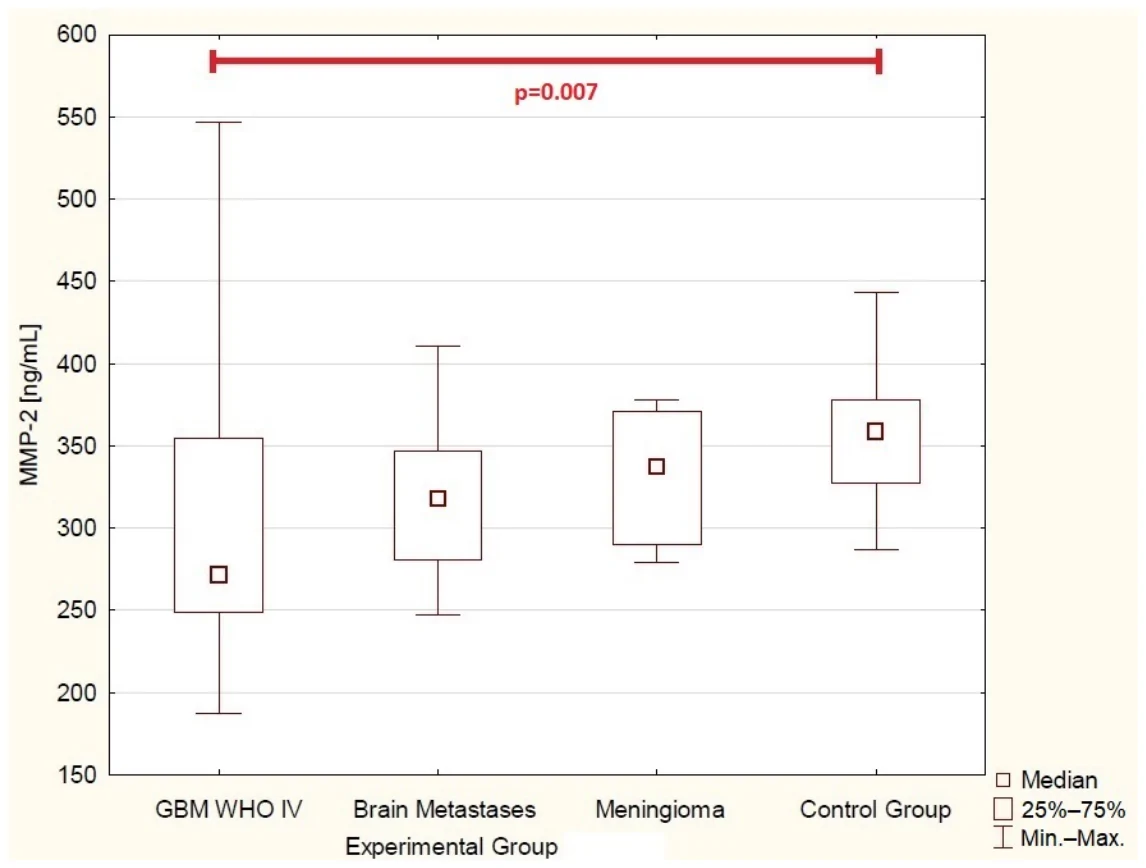
Median [interquartile range, IQR] serum concentration of matrix metalloproteinase-2 (MMP-2) in each group. The global between-group effect-size estimate was η^2^ = 0.22. The displayed pairwise *p*-value is provided as complementary inferential information.

**Figure 2:**
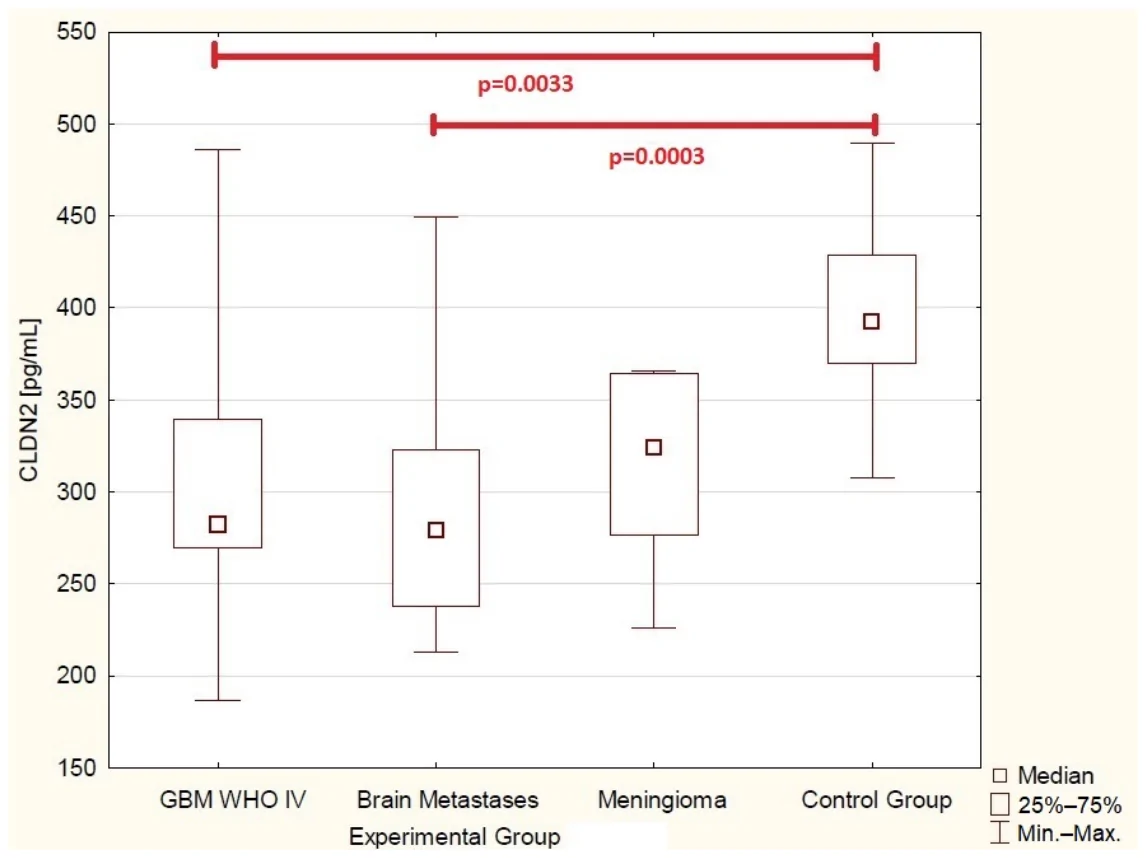
Median [interquartile range, IQR] serum concentration of claudin-2 (CLDN2) in each group. The global between-group effect-size estimate was η^2^ = 0.35. The displayed pairwise *p*-values is provided as complementary inferential information.

Exploratory post hoc pairwise comparisons were used to identify which group contrasts contributed to the larger global effects observed for MMP-2 and CLDN2. Following Benjamini–Hochberg correction, MMP-2 concentrations were lower in the GBM group than in the surgical control group, whereas CLDN2 concentrations were lower in the GBM and brain metastases groups than in the surgical control group. Figure 1 and Figure 2 present these group comparisons. Data are expressed as medians with interquartile ranges (IQRs).

Serum 25(OH)D concentrations were comparable across the four study groups. The global effect-size estimate was small for MMP-9 (η^2^ = 0.05) and negligible for PDIA4 (η^2^ = 0.00), with no clear pairwise between-group differences observed for either marker.

Spearman’s rank correlation analysis was performed to assess relationships between circulating biomarkers within each study group. Only weak associations were observed between biomarkers, and these were not further interpreted.

**Figure 3:**
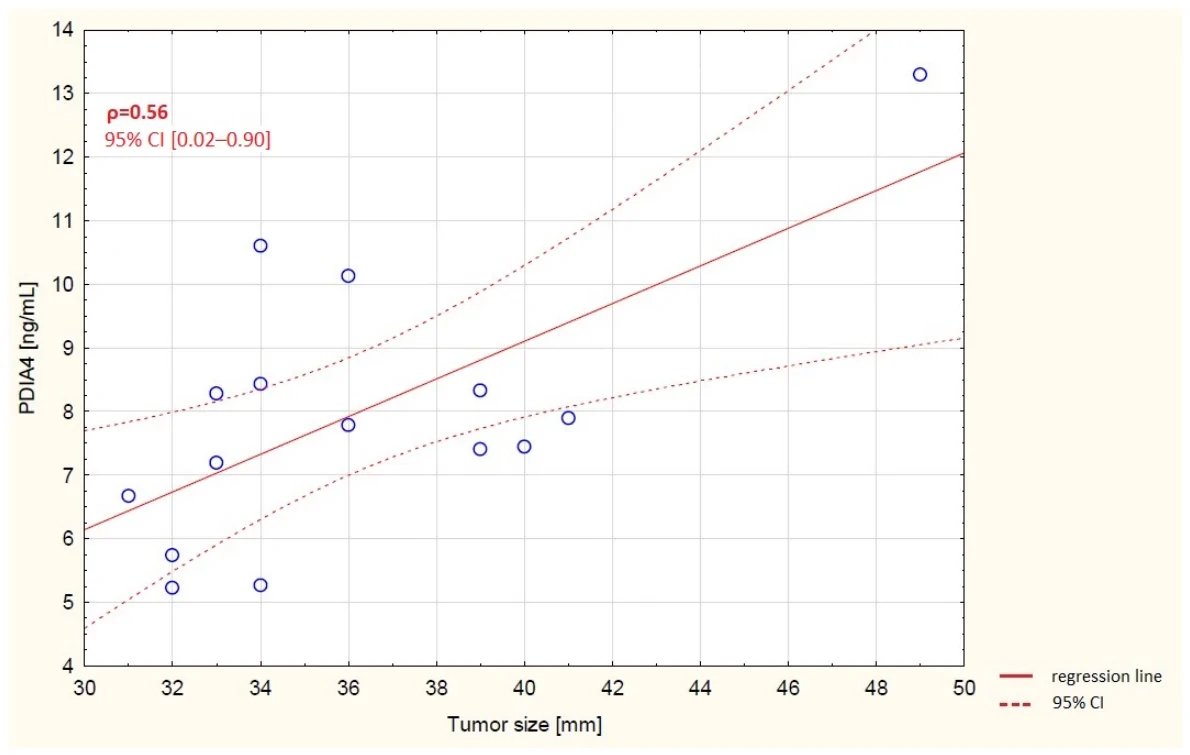
Correlation between protein disulfide isomerase A4 (PDIA4) levels and tumor size in the glioblastoma (GBM) WHO IV group. The plot shows individual data points, the regression line, the 95% confidence band, and Spearman’s rho with 95% confidence interval.

The relationship between serum biomarker concentrations and tumor size was analyzed separately within each tumor group. In the GBM group, serum PDIA4 concentration showed an exploratory moderate positive association with tumor size (Spearman’s rho = 0.56; 95% CI = 0.02–0.90) (Figure 3).

**Figure 4:**
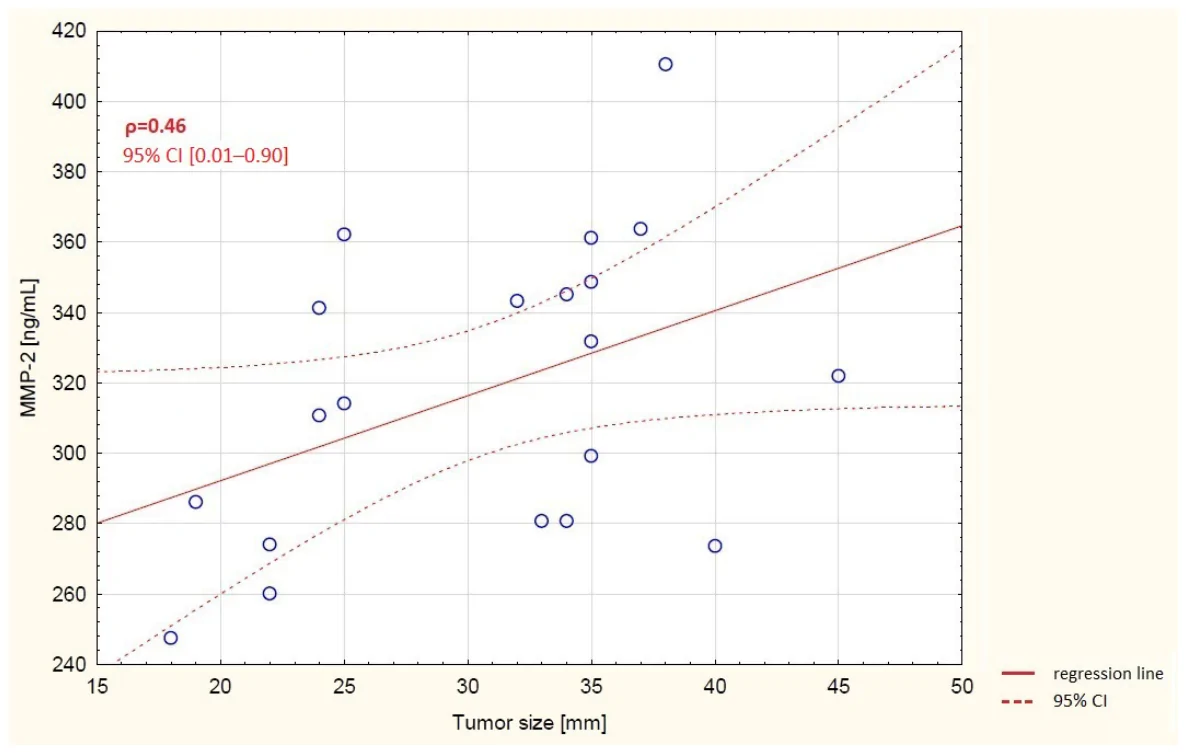
Correlation between matrix metalloproteinase-2 (MMP-2) levels and tumor size in the brain metastases group. The plot shows individual data points, the regression line, the 95% confidence band, and Spearman’s rho with 95% confidence interval.

In patients with brain metastases, serum MMP-2 concentration showed an exploratory weak-to-moderate positive association with tumor size; however, the wide confidence interval indicates substantial uncertainty around this estimate (Spearman’s rho = 0.46; 95% CI = 0.01–0.90) (Figure 4).

No relevant associations with tumor size were observed for the remaining biomarkers.

## 4. Discussion

This study was designed as a pilot investigation aimed at further exploring the role of selected markers of ECM remodeling and BBB integrity in the pathogenesis of brain tumors. Dysregulation of these processes, including both the activation of proteases involved in ECM degradation and dysfunction of tight junctions regulating BBB permeability, is widely recognized as a key mechanism promoting the growth, infiltration, and progression of central nervous system malignancies. Therefore, the assessment of circulating biomarkers reflecting these phenomena may provide valuable insights into tumor biology as well as clinical behavior.

In our study, we analyzed serum concentrations of PDIA4, MMP-2, MMP-9, and CLDN2 in patients with various types of brain tumors and evaluated their associations with tumor size. All patients participating in this study had vitamin D deficiency. Vitamin D deficiency is common in the adult population, particularly at our latitude [31]. Selecting such a cohort reflects a real-world clinical problem. Moreover, vitamin D status influences the inflammatory response and connective tissue metabolism and may consequently modify the levels of proteolysis and tissue remodeling markers (including MMPs) [32]. Restricting inclusion to individuals with deficiency reduces biological heterogeneity and limits the risk that the observed differences are driven by baseline differences in vitamin D status. The analyzed cohorts comprised patients with malignant primary brain tumors (GBM), patients with secondary brain tumors (brain metastases), and patients with meningioma, a benign, extra-axial lesion causing the compression of brain tissue. Biomarker levels were also assessed in a control group consisting of patients undergoing neurosurgical procedures for degenerative spine disease. Given the exploratory design, interpretation focused on the magnitude and pattern of the global between-group effect-size estimates rather than on statistical significance alone. The largest estimates were observed for CLDN2 (η^2^ = 0.35) and MMP-2 (η^2^ = 0.22), whereas smaller estimates were observed for MMP-9 (η^2^ = 0.05) and PDIA4 (η^2^ = 0.00). Exploratory pairwise comparisons indicated lower MMP-2 concentrations in patients with GBM than in the surgical control group. This direction of difference was unexpected when considered in the context of previously reported tissue-level MMP-2 expression. Lower CLDN2 concentrations were also observed in patients with GBM and brain metastases than in the surgical control group. Exploratory positive associations were also observed between tumor size and PDIA4 in patients with GBM and between tumor size and MMP-2 concentrations in patients with brain metastases.

Matrix metalloproteinases—particularly MMP-2 and MMP-9—are widely described as enzymes that facilitate tumor invasion by degrading components of the basement membrane and extracellular matrix. In high-grade tumors such as GBM, numerous studies have demonstrated increased MMP-2/MMP-9 expression in tumor tissue, as well as associations with a more aggressive phenotype and poorer prognosis [33,34,35,36,37]. Moreover, circulating MMP measurements have been investigated as potential blood-based biomarkers in glioma, particularly MMP-9, and also as part of multi-marker panels including MMP-2 [38,39]. The large global between-group effect-size estimate for MMP-2 (η^2^ = 0.22) was accompanied by lower serum concentrations in patients with GBM than in the surgical control group. This observation contrasts with tissue-based data indicating increased MMP-2 expression in high-grade gliomas and suggests that circulating MMP-2 levels may not directly mirror intratumoral MMP-2 activity. Accordingly, the lower serum MMP-2 concentrations observed in the GBM group compared with the surgical control group should not be interpreted solely as a tumor-specific decrease. They may also partly reflect elevated proteolytic activity within the degenerative spine disease control cohort.

First, the compartmentalization of biological processes (tumor tissue vs. peripheral circulation) should be considered. In GBM, MMP-2 may be produced intensively within the local tumor microenvironment, which includes tumor cells, glioma-associated macrophages/microglia, endothelial cells, extracellular matrix components, and extracellular vesicle-mediated intercellular communication. These local interactions may influence protease expression, matrix remodeling, angiogenesis, and immune modulation, while being only partially reflected in peripheral blood measurements [40,41]. Therefore, the passage of MMP-2 into the peripheral bloodstream may be limited and variable. This may result from several mechanisms: (i) partial preservation or heterogeneous dysfunction of the blood–brain barrier [42,43], (ii) local activation and retention of the enzyme within the extracellular matrix [44], and (iii) differences between the proenzyme and active enzyme forms, as well as modulation of activity by endogenous inhibitors (e.g., TIMPs) [45]. Consequently, increased tissue expression of MMP-2 does not necessarily translate linearly into its serum concentration. This is consistent with observations that serum-based findings on MMPs in CNS tumors can be inconsistent and depend, among other factors, on the choice of comparator groups and methodology [46].

Second, the observed differences may also have been influenced by clinical factors typical of the GBM disease course. Patients with GBM frequently receive glucocorticoids because of peritumoral edema and increased intracranial pressure, and systemic immune dysregulation in GBM is well documented [47,48]. In our cohort, most patients with malignant tumors received perioperative corticosteroids, which limited our ability to separate tumor-related effects from treatment-related modulation of circulating inflammatory and proteolytic markers. Corticosteroid exposure, tumor necrosis, peritumoral edema, infections, thromboembolic events, and other systemic complications may all generate non-specific inflammatory or proteolytic signals [49,50,51]. These factors may obscure tumor-specific changes in serum MMP-2, particularly because MMPs are sensitive to inflammation, tissue remodeling, and therapeutic interventions.

CLDN2 showed the largest global between-group effect-size estimate (η^2^ = 0.35), with lower circulating concentrations in patients with GBM and brain metastases than in the surgical control group. This finding should be interpreted cautiously, as data specifically addressing serum CLDN2 in malignant brain tumors remain limited. Most studies on tight-junction remodeling in CNS tumors have focused on CLDN5, occludin, ZO-1, and other endothelial junctional proteins rather than CLDN2 [52,53]. Therefore, serum CLDN2 should not be regarded as a direct marker of blood–brain barrier or blood–tumor barrier integrity.

Nevertheless, the observed differences may be considered within the broader context of tight-junction remodeling in intracranial tumors. In GBM, glioma-derived factors have been shown to increase endothelial permeability and reduce the expression of tight-junction proteins, including claudins and occludin [53,54]. Tissue-based studies in high-grade gliomas have also demonstrated altered expression of tight-junction proteins, particularly CLDN1, CLDN5, and occludin [53]. In addition, MMP-2 and MMP-9 may promote the degradation of tight-junction proteins and contribute to increased BBB permeability [55].

Similar mechanisms may be relevant in brain metastases, where colonization of the CNS is associated with remodeling of the BBB and formation of a more permeable blood–tumor barrier. In experimental models, altered expression and localization of tight-junction proteins, particularly CLDN5 and occludin, have been shown to affect barrier permeability and tumor cell invasion [56,57,58]. Although these data do not directly explain circulating CLDN2 concentrations, they support the broader concept that malignant brain tumors are associated with vascular barrier remodeling.

Lower CLDN2 concentrations in patients with GBM and brain metastases may therefore reflect altered tight-junction protein turnover, enhanced degradation, or systemic differences between groups, rather than a direct relationship between tumor presence and CLDN2 expression. CLDN2 may undergo proteolytic cleavage under inflammatory conditions and can be removed from the membrane by endocytosis and targeted for autophagy-dependent lysosomal degradation [59,60,61]. The role of CLDN2 in cancer is also tissue- and context-dependent: CLDN2 has been implicated in colorectal cancer progression and metastatic growth, whereas in renal clear cell carcinoma, it may exert an inhibitory effect on tumor progression through YAP-related mechanisms [62,63]. This finding should therefore be regarded as exploratory and requires validation in larger cohorts with parallel tissue-level assessment.

Consistent with the small global effect-size estimate for MMP-9 (η^2^ = 0.05) and the negligible estimate for PDIA4 (η^2^ = 0.00), neither marker showed clear pairwise between-group differences in this pilot cohort. These effect-size should be interpreted cautiously given the modest sample size of the individual groups (GBM, *n* = 15; brain metastases, *n* = 20; meningioma, *n* = 8; surgical controls, *n* = 19), inter-individual variability, and the influence of perioperative clinical factors. This pattern may suggest that, despite their reported tissue-level relevance in gliomas and other malignancies [35,36,64], PDIA4 and MMP-9 may not generate a consistent peripheral-blood signal in this setting. These observations provide a preliminary rationale for evaluating multi-marker panels and highlight the need for carefully controlled sampling in future studies.

Data directly addressing associations between circulating PDIA4 or MMP-2 and tumor size in these tumor entities remain scarce. Although such data remain limited, both findings are biologically plausible. PDIA4 has been shown to promote glioblastoma progression and to contribute to angiogenesis-related mechanisms in GBM [12,15], whereas MMP-2 is a recognized mediator of tumor invasion, extracellular matrix remodeling, vascular patterning, and tumor-associated neovascularization [16,17,18,44,45]. Given the small subgroup sizes and wide 95% confidence intervals, these associations should be regarded as imprecise exploratory estimates rather than robust evidence of relationships with tumor size.

The results should be interpreted in light of several limitations. First, this study has inherent limitations related to its exploratory pilot design and modest sample size. No formal a priori sample size calculation was performed, and this study was therefore not powered for confirmatory statistical inference. Accordingly, the effect-size estimates represent the principal quantitative outputs of this pilot study and should be interpreted as preliminary and hypothesis-generating rather than confirmatory. Because no predictive or classification model was developed, cross-validation was not applicable to the present analysis. An independent external validation cohort was not available, and the modest sample size did not permit reliable multivariable adjustment for potential clinical and perioperative confounders. Accordingly, the observed effect-size estimates should be considered preliminary and require confirmation in larger, independent, prospectively characterized cohorts. Although the Benjamini–Hochberg procedure was applied to control the false discovery rate in the context of multiple testing, this approach reduces but does not eliminate the possibility of type I error.

Second, measurements were restricted to serum concentrations without parallel tissue-level validation, which constrains mechanistic inference. Future studies should combine circulating biomarker assessment with tissue-level analyses to better determine whether serum concentrations reflect intratumoral expression or activity.

Third, perioperative corticosteroid exposure represents another important limitation. In patients undergoing surgery for intracranial tumors, corticosteroids are commonly used as part of routine clinical management, particularly in the presence of peritumoral edema or mass effect. Corticosteroids may influence inflammatory, endothelial, tight-junction-related, and proteolytic pathways, including MMP-related signaling, and may therefore affect circulating biomarker concentrations. However, detailed data regarding corticosteroid dose, duration, timing, and cumulative exposure were not systematically recorded in the study cohort. Therefore, a reliable subgroup analysis comparing corticosteroid-exposed and non-exposed patients could not be performed. Future studies should prospectively collect detailed corticosteroid exposure data and stratify or adjust analyses accordingly.

Fourth, another important limitation concerns the use of patients with degenerative spine disease as the surgical control group. This group was selected to provide a clinically comparable neurosurgical cohort rather than a healthy volunteer baseline. However, degenerative spine disease and intervertebral disk degeneration are associated with chronic inflammatory processes, extracellular matrix remodeling, and increased expression or activity of matrix-degrading enzymes, including MMP-related pathways [65,66,67]. Therefore, the control group should not be interpreted as a healthy baseline group. These biological characteristics may have contributed to the higher circulating MMP-2 concentrations observed in controls and may have confounded between-group comparisons. Future studies should include healthy controls and/or carefully selected disease-control cohorts to better distinguish tumor-related biomarker patterns from non-specific inflammatory and proteolytic signals.

Despite these limitations, the differing effect-size patterns observed across the analyzed biomarkers suggest that markers representing distinct biological axes may provide complementary information in future multi-marker studies.

In summary, markers with a well-established tissue-level role in tumor biology may exhibit a different “signature” in the peripheral circulation, likely reflecting compartmentalization (tumor vs. blood), variable blood–brain barrier permeability, and the influence of clinical factors (including perioperative management and inflammatory status).

The principal quantitative findings of this exploratory pilot study were the global between-group effect-size estimates. The largest estimates were observed for CLDN2 (η^2^ = 0.35) and MMP-2 (η^2^ = 0.22), whereas the estimate was small for MMP-9 (η^2^ = 0.05) and negligible for PDIA4 (η^2^ = 0.00). Exploratory pairwise comparisons indicated lower circulating MMP-2 concentrations in patients with GBM than in the surgical control group and lower CLDN2 concentrations in patients with GBM and brain metastases than in the surgical control group. Exploratory positive associations were also observed between PDIA4 and tumor size in GBM and between MMP-2 and tumor size in brain metastases. Given the modest sample size and absence of independent validation, these findings should be considered preliminary and hypothesis-generating rather than robust diagnostic or prognostic evidence. Nevertheless, the effect-size estimates may inform biomarker prioritization and sample-size planning for larger prospective validation studies and provide a rationale for further evaluation of multi-marker blood-based approaches.

## Figures and Tables

**Table 1:** Baseline characteristics.

	Experimental Group			Control Group	
	GBM Who IV (*n* = 15)	Brain Metastases (*n* = 20)	Meningioma (*n* = 8)	Spinal Disk Disease (*n* = 19)	*p*-Value
Females, *n* (%)	4 (26.7)	9 (45.0)	5 (62.5)	12 (63.2)	0.156 (*)
Males, n (%)	11 (73.3)	11 (55.0)	3 (37.5)	7 (36.8)	
Age, Mean (±SD)	62.7 (±11.3)	63.6 (±9.4)	65.1 (±10.1)	67.1 (±13.5)	0.672 (**)
VIT. D3 [ng/mL], Median (IQR)	3.72 (3.32–4.84)	3.99 (3.57–4.30)	3.97 (3.75–4.15)	4.19 (4.07–4.75)	0.188 (***)

Between-group differences were evaluated using the chi-square test (*) for categorical variables, analysis of variance ANOVA (**) for parametric data, and the Kruskal–Wallis test (***) for non-parametric data Abbreviations: GBM, glioblastoma; SD, standard deviation; IQR, interquartile range.

**Table 2:** Global between-group effect-size estimates and Kruskal–Wallis test results. Abbreviations: PDIA4, protein disulfide isomerase A4; MMP-2, matrix metalloproteinase-2; MMP-9, matrix metalloproteinase-9; CLDN2, claudin-2; η^2^, eta-squared effect size.

	η^2^	Effect Size Interpretation	H (3, *N* = 62)	*p*-Value
PDIA4 [ng/mL]	0.00	Negligible effect	1.97	0.579
MMP-9 [ng/mL]	0.05	Small effect	6.00	0.111
MMP-2 [ng/mL]	0.22	Large effect	15.58	0.001
CLDN2 [pg/mL]	0.35	Large effect	23.29	<0.001

## Data Availability

The datasets generated and analyzed during the current study are not publicly available due to ethical and privacy restrictions but are available from the corresponding author upon reasonable request.

## Supplementary Materials

The following supporting information can be downloaded at https://www.mdpi.com/article/10.3390/jcm15166192/s1, The completed STROBE checklist for cross-sectional studies is provided as Supplementary File S1. The simplified patient selection and diagnostic classification diagram is provided as Supplementary Figure S1.

## Abbreviations

The following abbreviations are used in this manuscript:

BBB	blood–brain barrier
BTB	blood–tumor barrier
CLDN2	claudin-2
CNS	central nervous system
ECM	extracellular matrix
ER	endoplasmic reticulum
GBM	glioblastoma
MMP	matrix metalloproteinase
MMP-2	matrix metalloproteinase-2
MMP-9	matrix metalloproteinase-9
PDIA4	protein disulfide isomerase A4
UPR	unfolded protein response
VEGF	vascular endothelial growth factor
XBP1	X-box binding protein 1
YAP	Yes-associated protein
