# Supplementary material for: Circulating PDIA4, MMP-2, MMP-9, and Claudin-2 in Vitamin D-Deficient Patients with Brain Tumors: An Exploratory Clinical Pilot Study
Source: J Clin Med. 2026 Aug 10;15(16):6192. doi: 10.3390/jcm15166192 (PMC13513073; doi:10.3390/jcm15166192)
Supplement: Supplementary file 1 [file jcm-15-06192-s001.zip › Figure S1.pdf]

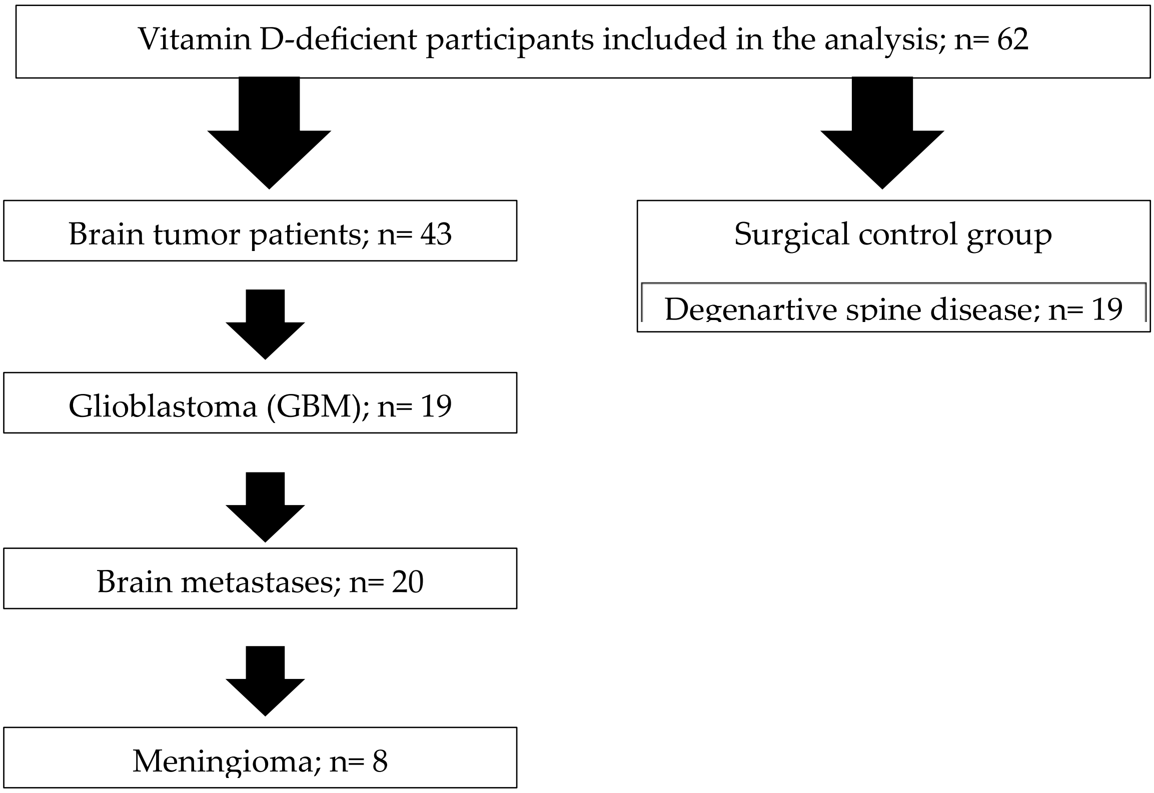

Figure S1. Simplified patient selection and diagnostic classification diagram. Vitamin D-deficient participants included in the analysis (n = 62) comprised brain tumor patients (n = 43), including glioblastoma (n = 15), brain metastases (n = 20), and meningioma (n = 8), and a surgical control group with degenerative spine disease (n = 19).
