# Supplementary material for: Circulating PDIA4, MMP-2, MMP-9, and Claudin-2 in Vitamin D-Deficient Patients with Brain Tumors: An Exploratory Clinical Pilot Study
Source: J Clin Med. 2026 Aug 10;15(16):6192. doi: 10.3390/jcm15166192 (PMC13513073; doi:10.3390/jcm15166192)
Supplement: Supplementary file 1 [file jcm-15-06192-s001.zip › Supplementary File STROBE.pdf]

Supplementary File S1

STROBE Statement—Checklist of Items That Should Be Included in Reports of Cross-Sectional Studies

Manuscript: “Circulating PDIA4, MMP-2, MMP-9, and Claudin-2 in Vitamin D-Deficient Patients with Brain Tumors: An Exploratory Clinical Pilot Study”

Section / topic	Item No.	STROBE recommendation	Reported on page/section of the revised manuscript
Title and abstract	**1(a)**	Indicate the study’s design with a commonly used term in the title or the abstract.	Partially reported. The title and Abstract, pp. 1–2, identify the study as an exploratory pilot study. Section 2.1, p. 4, describes it as observational and non-interventional. The cross-sectional design is not explicitly named in the title or abstract.
Title and abstract	**1(b)**	Provide in the abstract an informative and balanced summary of what was done and what was found.	Abstract, p. 2.
**Introduction**			
Background/rationale	**2**	Explain the scientific background and rationale for the investigation being reported.	Introduction, pp. 2–4.
Objectives	**3**	State specific objectives, including any prespecified hypotheses.	Final paragraph of the Introduction, p. 4. Primary and secondary objectives are stated.
**Methods**			
Study design	**4**	Present key elements of study design early in the paper.	Sections 2.1–2.2, pp. 4–5. The study is described early as an observational, non-interventional exploratory pilot study conducted in a single neurosurgical center. Participants were assigned to predefined clinical groups, and serum biomarkers were assessed using perioperative blood samples.
Setting	**5**	Describe the setting, locations, and relevant dates, including periods of recruitment, exposure, follow-up, and data collection.	Sections 2.1–2.2, pp. 4–5. The study setting, single-center location, study period (September 2021–February 2022), and perioperative blood collection are described.
Participants	**6**	Give the eligibility criteria, and the sources and methods of selection of participants.	Section 2.2, pp. 4–5. The source population, study period, principal eligibility criteria, diagnostic classification, and control-group criteria are described.
Variables	**7**	Clearly define all outcomes, exposures, predictors, potential confounders, and effect modifiers. Give diagnostic criteria, if applicable.	Final paragraph of the Introduction and Sections 2.2–2.4, pp. 4–5. The variables of interest, diagnostic groups, and the criterion for vitamin D deficiency are described.
Data sources/measurement	**8***	For each variable of interest, give sources of data and details of methods of assessment (measurement). Describe comparability of assessment methods if there is more than one group.	Sections 2.2–2.3, pp. 4–5. Tumor groups were classified according to postoperative histopathological diagnosis. Venous blood collection, serum processing and storage, and ELISA measurement procedures are described and were applied across the study groups.
Bias	**9**	Describe any efforts to address potential sources of bias.	Sections 2.2–2.3, pp. 4–5, and Discussion, pp. 10–12. Restriction of the cohort to vitamin D-deficient participants was intended to reduce heterogeneity related to vitamin D status. Standardized serum collection, processing, storage, and ELISA procedures are described. Potential selection and survivorship bias, perioperative corticosteroid exposure, serum-only assessment, and limitations of the surgical control group are discussed.
Study size	**10**	Explain how the study size was arrived at.	Section 2.2, pp. 4–5, and Discussion, p. 11. The study included 62 participants. No formal a priori sample-size calculation was performed because of the exploratory pilot nature of the study.
Quantitative variables	**11**	Explain how quantitative variables were handled in the analyses. If applicable, describe which groupings were chosen and why.	Section 2.4, p. 5, and Table 1, p. 6. Quantitative variables were summarized using mean (SD) or median (IQR), as appropriate. Biomarker concentrations and tumor size were analyzed as continuous variables, and non-parametric methods were used for biomarker comparisons and correlations.
Statistical methods	**12(a)**	Describe all statistical methods, including those used to control for confounding.	Section 2.4, p. 5, and Table 1, p. 6. Normality was assessed using the Shapiro–Wilk test. Between-group comparisons were performed using the chi-square test, analysis of variance, or the Kruskal–Wallis test with post hoc comparisons, as appropriate. Effect sizes were expressed as η^2, multiple comparisons were controlled using the Benjamini–Hochberg procedure, and associations were assessed using Spearman’s rank correlation with bootstrap confidence intervals.

Section / topic	Item No.	STROBE recommendation	Reported on page/section of the revised manuscript
Statistical methods	**12(b)**	Describe any methods used to examine subgroups and interactions.	Section 2.4, p. 5, and Results, pp. 8–9. Spearman's rank correlations were used to assess associations between serum biomarkers and tumor size separately within each diagnostic tumor group.
Statistical methods	**12(c)**	Explain how missing data were addressed.	Complete data were available for all variables included in the reported analyses. No imputation was required.
Statistical methods	**12(d)**	If applicable, describe analytical methods taking account of sampling strategy.	Not applicable. No complex sampling design or sampling weights were used.
Statistical methods	**12(e)**	Describe any sensitivity analyses.	No sensitivity analyses were performed.
**Results**			
Participants	**13(a)***	Report numbers of individuals at each stage of study—e.g., numbers potentially eligible, examined for eligibility, confirmed eligible, included in the study, and analysed.	Sections 2.2 and 3, pp. 4–6. A total of 62 participants were included and analyzed: patients with GBM (n = 15), brain metastases (n = 20), meningioma (n = 8), and surgical controls (n = 19).
Participants	**13(b)***	Give reasons for non-participation at each stage.	Section 2.2, pp. 4–5. Individuals who did not meet the predefined group-specific eligibility criteria were not enrolled
Participants	**13(c)***	Consider use of a flow diagram.	No flow diagram was used. Final cohort allocation and group sizes are reported in Sections 2.2 and 3, pp. 4–6, and Table 1.
Descriptive data	**14(a)***	Give characteristics of study participants (e.g., demographic, clinical, social) and information on exposures and potential confounders.	Table 1, p. 6, and Section 2.2, pp. 4–5. Participant numbers, sex, age, serum 25(OH)D concentration, and clinical-group characteristics are reported
Descriptive data	**14(b)***	Indicate number of participants with missing data for each variable of interest.	No missing data were present for the variables included in the reported analyses. Tumor-size assessment was applicable only to participants in the tumor groups.
Outcome data	**15***	Report numbers of outcome events or summary measures.	Table 2 and Figures 1–4, pp. 6–9. Global Kruskal–Wallis statistics and effect sizes are reported for PDIA4, MMP-2, MMP-9, and CLDN2. Group distributions are presented for MMP-2 and CLDN2, and correlation estimates with 95% confidence intervals are reported for the selected associations with tumor size.
Main results	**16(a)**	Give unadjusted estimates and, if applicable, confounder-adjusted estimates and their precision (e.g., 95% confidence interval). Make clear which confounders were adjusted for and why they were included.	Table 2, p. 6; Figures 1–2, p. 7; and Figures 3–4, pp. 8–9. Unadjusted global η^2 effect estimates and p-values, Benjamini–Hochberg-adjusted pairwise p-values, and Spearman's rho values with bootstrap 95% confidence intervals are reported. No confounder-adjusted estimates were calculated
Main results	**16(b)**	Report category boundaries when continuous variables were categorized.	Section 2.2, p. 5. Vitamin D deficiency is defined as serum 25(OH)D <20 ng/mL (<50 nmol/L); biomarker concentrations and tumor size are analyzed as continuous variables.
Main results	**16(c)**	If relevant, consider translating estimates of relative risk into absolute risk for a meaningful time period.	Not applicable. The study did not estimate relative or absolute risks.
Other analyses	**17**	Report other analyses done—e.g., analyses of subgroups and interactions, and sensitivity analyses.	Results, pp. 8–9. Within-group biomarker correlations and diagnostic-group-specific associations between biomarkers and tumor size are reported.
**Discussion**			
Key results	**18**	Summarise key results with reference to study objectives.	Discussion, pp. 9–12, including the opening and concluding summary paragraphs.
Limitations	**19**	Discuss limitations of the study, taking into account sources of potential bias or imprecision. Discuss both direction and magnitude of any potential bias.	Discussion, pp. 10–12. The manuscript addresses pilot sample size and imprecision, multiple testing, absence of tissue-level validation, corticosteroid exposure, selection and survivorship considerations, and the characteristics of the surgical control group.
Interpretation	**20**	Give a cautious overall interpretation of results considering objectives, limitations, multiplicity of analyses, results from similar studies, and other relevant evidence.	Discussion, pp. 9–12. The findings are interpreted in relation to the study objectives, methodological limitations, multiplicity, and the existing tissue-level and circulating-biomarker literature.
Generalisability	**21**	Discuss the generalisability (external validity) of the study results.	Section 2.2, pp. 4–5, and Discussion, pp. 11–12. The manuscript describes the surgically treated, vitamin D-deficient neurosurgical cohort and the need for validation in larger, well-controlled cohorts.

Section / topic	Item No.	STROBE recommendation	Reported on page/section of the revised manuscript
**Other information**			
Funding	**22**	Give the source of funding and the role of the funders for the present study and, if applicable, for the original study on which the present article is based.	Funding statement, p. 13: “This research received no external funding.”

* *Cross-sectional study-specific item.*

Checklist source: STROBE Statement—Checklist of items that should be included in reports of cross-sectional studies (STROBE Initiative; www.strobe-statement.org).
